# Genetically Modified Porcine Mesenchymal Stem Cells by Lentiviral Tbx18 Create a Biological Pacemaker

**DOI:** 10.1155/2019/3621314

**Published:** 2019-11-07

**Authors:** Yannan Hu, Ning Li, Liang Liu, Hao Zhang, Xiang Xue, Xin Shao, Yu Zhang, Xilong Lang

**Affiliations:** ^1^Department of Cardiothoracic Surgery, Changhai Hospital, Second Military Medical University, Shanghai, China; ^2^Department of Thoracic Surgery, Changzhou TCM Hospital, Changzhou, China; ^3^Department of Cardiothoracic Surgery, The Affiliated Second Hospital, Suzhou University, Suzhou, China

## Abstract

**Background:**

Tbx18 is a vital transcription factor involved in embryonic sinoatrial node (SAN) formation process but is gradually vanished after birth. Myocardial injection of lentiviral Tbx18 converts cardiomyocytes into pacemaker-like cells morphologically and functionally. In this *in vitro* and *in vivo* study, genetical modification of porcine bone mesenchymal stem cells (BMSCs) by recapturing the Tbx18 expression creates a biological pacemaker which was examined.

**Methods:**

The isolated porcine BMSCs were transfected with lentiviral Tbx18, and the induced pacemaker-like cells were analyzed using real-time polymerase chain reaction and western blotting to investigate the efficiency of transformation. Then, the induced pacemaker-like cells were implanted into the right ventricle of the SAN dysfunction porcine model after the differentiation process. Biological pacemaker activity and ectopic pacing region were tested by an electrocardiograph (ECG) monitor.

**Results:**

The isolated porcine BMSCs expressed specific surface markers of stem cells; meanwhile, the expression of myocardial markers was upregulated significantly after lentiviral Tbx18 transfection. The porcine SAN dysfunction model was constructed by electrocoagulation using a surgical electrotome. The results showed that the mean heart beat (HR) of BMSCs-Tbx18 was significantly higher than that of BMSCs-GFP. An ectopic pacing region was affirmed into the right ventricle by ECG after implantation of BMSCs-Tbx18.

**Conclusion:**

It was verified that Lenti-Tbx18 is capable of transducing porcine BMSCs into pacemaker-like cells. Genetically modified porcine BMSCs by lentiviral Tbx18 could create a biological pacemaker. However, further researches in large-scale animals are required to rule out unexpected complications prior to application in clinical practice.

## 1. Introduction

At present, electronic pacemakers have become the standard treatment for bradyarrhythmia, including sick sinus syndrome (SSS) and atrioventricular block. It is reported that SSS is the predominant indication for pacemaker implantation in the United States, and the incidence of SSS increases with age. Hence, the number of these patients will increase sharply over the next 50 years and inevitably burden the medical budget [1]. The application of pacemakers has significantly improved the life quality of these patients. However, it should be noted that implanted electronic pacemakers can only alleviate the symptoms related to bradyarrhythmia while accompanied with some complications, including bleeding, infection, and battery exhaustion. In addition, pacemakers merely guarantee the basic physiological needs for these patients on account of that the pacing parameters are usually preset as certain values, which can hardly meet the requirements under various conditions.

Gene and cell therapies spark the development of biological pacemakers, which may address such limitations [2]. It is well known that the sinoatrial node (SAN) is the origin of cardiac electrical activity and sets the rhythm for the heart. SAN is different from other cardiac tissue in that its electric potential can be automatically depolarized at hyperpolarization. Therefore, a biological pacemaker can be achieved by restoring pacing ability of SAN or creating an ectopic pacing region.

One prevalent approach to acquire a biological pacemaker is genetic reprogramming of ventricular myocytes by a viral vector, including the embryonic transcription factors related to SAN formation and ion channel-associated proteins, to create an ectopic pacing region [3]. For example, the T-box (Tbx) gene family, including Tbx3 and Tbx18, plays an important role in the embryonic formation of SAN, and the short stature homeobox transcription factor 2 (Shox2) is also involved in this developmental process [4–6]. Moreover, ion channel-associated proteins, mainly hyperpolarization-activated cyclic nucleotide-gated channel 4 (HCN4), are related to the diastolic depolarization process of SAN [7]. *In vitro* and *in vivo* studies of gene manipulation for rodent neonatal rat cardiomyocytes to restore the ectopic pacing region by overexpressing embryonic transcription factors and ion channel-associated proteins have been proved to be successful [8–12]. Kapoor et al. have proved that Tbx18 was the most effective transcription factor with the ability to transform neonatal ventricular myocytes into SAN-like cells by transducing a panel of transcription factors individually, including Shox2, Tbx3, Tbx5, Tbx18, and Tbx20 [11]. It is worth mentioning that these reconstructed cells are similar to native SAN cells in both morphology and physiological automaticity [11]. Tbx18 has also been used to induce pacemaker-like cells from somatic myocytes *in situ* in a large-scale animal model of complete heart block [13, 14]. However, adenovirus-based genetic manipulation cannot maintain its function more than 2-4 weeks, which hinders its clinical feasibility.

Another avenue to biological pacing is gene- and cell-based hybrid treatment, which can be achieved by loading stem cells with the pacemaker gene for prolonged gene expression duration. Stem cells are optimal candidate carriers for their self-renewal and multipotent differentiation ability. Phenotypic transformation of BMSCs into pacemaker-like cells mediated by Tbx18 has been verified in the mRNA and protein level [15–17]. Besides, both Shox2 and Tbx3 have also been confirmed to enhance the differentiation efficacy of stem cells into pacemaker-like cells [18–20]. However, those studies in large-scale animal models are limited.

To the best of our knowledge, previous investigations mainly focused on inducing pacemaker-like cells via genetic reprogramming of somatic cardiomyocytes or evaluating the possibility for acquiring pacemaker-like cells by oriented differentiation from stem cells. In this article, we created a biological pacemaker by recapturing the Tbx18 expression of BMSCs *in vitro* and in a large-scale animal model. To this end, this study may provide a new genetic and cellular therapy for the treatment of bradyarrhythmia.

## 2. Materials and Methods

### 2.1. Porcine BMSC Isolation and Cell Culture

Porcine bone marrow was harvested aseptically into a 15 ml conical tube and suspended in 5 ml phosphate-buffered solution (PBS). The buffy coat was isolated by centrifugation (1500 rpm, 20 min) using 5 ml Ficoll (GE Healthcare, USA). The purified cells were collected and washed twice in sterile PBS [21]. The supernatant was removed after centrifugation, and then, the cells were resuspended and planked into 6-well plates with proper cell density. BMSCs were cultured in Dulbecco's modified Eagle's medium (Gibco, USA) supplemented with 100 U/ml penicillin (Gibco, USA), 100 *μ*g/ml streptomycin (Gibco, USA), and 10% FBS (Gibco, USA) at 37°C under a humidified 5% carbon dioxide incubator. The culture medium was refreshed every 2 days.

### 2.2. Construction of the Tbx18 Lentiviral Vector

PLenti-CMV-EGFP (Lenti-GFP) (OBiO Tech Co. Ltd., Shanghai, China) was digested using EcoR I and BamH I. The ORF sequence of the human Tbx18 gene (OBiO Tech Co. Ltd., Shanghai, China) was amplified using polymerase chain reaction (PCR). A restriction enzyme reaction procedure was performed before gel extraction. The digested gene fragment and vector were integrated to form pLenti-CMV-Tbx18-EGFP (Lenti-Tbx18), which was then transferred into competent DH5*α* cells (OBiO Tech Co. Ltd., Shanghai, China). Positive clones were identified by sequencing. Bacteria were incubated in a shaker at 37°C overnight in LB culture medium. Recombinant plasmids were extracted using a Plasmid Midi Preparation Kit (Beijing ComWin Biotech Co. Ltd., Beijing, China). HEK 293T cells were transfected with Lenti-Tbx18 and backbone vectors using Lipofectamine 2000 (Invitrogen, USA). The Lenti-GFP was packaged as a control. The supernatant was harvested after viral amplification. The acquired Lenti-Tbx18 and Lenti-GFP were stored at -80°C.

### 2.3. BMSCs Transfected with Tbx18

When BMSCs reached 70-80% confluency, lentiviral Tbx18 was used to transfect the cells at a multiplicity of infection (MOI) of 100. These transfected BMSCs were used as the experimental group; meanwhile, the BMSCs treated with Lenti-GFP were treated as the control. The culture medium was replaced with fresh complete medium after 6 h transfection procedure. These cells were observed using a fluorescent microscope to determine the transfection rate after 24 h. At last, these cells were cultured for an additional 7 days for morphological observation.

### 2.4. Flow Cytometry Analysis

The isolated BMSCs were collected by trypsinization, centrifuged at 1200 rpm for 5 min, washed by PBS twice, and then transferred into 1.5 ml EP tubes. The supernatant was discarded after centrifugation. BMSCs were blocked by BSA (Thermo, USA) solution for 30 min. Then, BMSCs were incubated with primary antibodies CD29 (ab6124, 1 : 400), CD90 (ab23894, 1 : 100), CD34 (ab81289, 1 : 100), and CD45 (ab10558, 1 : 400) overnight at 4°C, respectively. BMSCs without incubation with the primary antibody were set as a negative control. Then, the corresponding FITC-labeled secondary antibodies were added for 1 h at room temperature in the dark. Cells were centrifuged at 1500 rpm for 5 min, and the supernatant was removed. Cells were washed with PBS twice and resuspended in PBS prior to analysis using a flow cytometer.

### 2.5. mRNA Real-Time Quantitative PCR (RT-qPCR)

Total RNA was extracted by a TRIzol Reagent kit (Takara, Japan) following the manufacturer's protocol. The concentration was determined by spectrophotometry at 260 nm. For mRNA quantification, 1 *μ*g of total RNA was reverse-transcribed to cDNA using the PrimeScript RT reagent kit (Takara, Japan). The SYBR Green RT-PCR Kit (Takara, Japan) and LightCycler 480 System (Roche, Switzerland) were used for quantitative RT-PCR analysis. The mRNA expression was normalized to the reference gene GAPDH for each cDNA sample. Amplification conditions were set as follows: 95°C of preincubation for 3 min, 40 cycles of denaturation at 95°C for 30 s, annealing at 62°C for 30 s, and final extension at 72°C for 30 s. All results were normalized to GAPDH expression levels and compared to the expression level at baseline by the 2^-ΔΔCt^ method. The primers used in this experiment are presented in Table 1.

### 2.6. Western Blotting

After each treatment, cells were washed with PBS and lysed in SDS buffer containing a protease inhibitor cocktail (1 : 100) on ice for 30 minutes. Total protein concentrations were evaluated using a protein assay kit (Beyotime Biotech, Shanghai, China). A cell lysate was separated using 10% SDS-PAGE and then transferred to polyvinylidene difluoride membranes. Subsequently, the membranes were blocked for 1 h at room temperature by incubation in TBST containing 5% (*w*/*v*) nonfat milk. Primary antibodies against HCN4 (ab32675, 1 : 5000), cTnI (ab47003, 1 : 500), *α*-SA (ab156302, 1 : 1000), Tbx18 (ab115262, 1 : 800), and GAPDH (Proteintech, 1 : 5000) were incubated overnight at 4°C. Finally, the membranes were incubated with corresponding secondary antibodies for 1 hour at room temperature and detected by an ECL Chemiluminescent Substrate Reagent Kit (Thermo, USA) and exposed to a film.

### 2.7. Immunofluorescence Staining

The myocardial tissue at the injection site was fixed with 4% paraformaldehyde. Then, a routine three-step protocol was conducted: sectioning, dewaxing, and hydrating tissues. Following permeabilization with 0.1% Triton X-100, the tissue was incubated with a primary anti-Tbx18 antibody (ab115262, 1 : 200) overnight at 4°C. A secondary antibody with green fluorescence was then used to detect Tbx18. We used 4′,6-diamidino-2-phenylindole (DAPI) to visualize the nuclei. The cells were observed under a fluorescence microscope.

### 2.8. Large-Scale Animal Experiment

Adult Yorkshire pigs (male, 40 kg) were managed with conformation to the “Shanghai Administration Rule of Laboratory Animal,” and the Institutional Animal Care and Use Committee approved the protocol of the Animal Care Center at the Second Military Medical University. Firstly, the pig was sedated by injection of midazolam (0.25 mg/kg), supplied with ketamine (8 mg/kg) intramuscularly; then, the pig was constrained on the animal operating table and endotracheally intubated. Electrocardiograph (ECG) leads were inserted into the 4 limbs subcutaneously and connected to the data acquisition (DAQ) system for continuous signal collection. Mechanical ventilation was performed for proper perioperative management and prevention of pneumothorax. Subsequently, anesthesia was maintained by propofol (4 mg/kg/h, intravenously). Right 4^th^ intercostal mini-incision was employed to provide straight access to the right heart and minimize operative wound. The pericardium was opened using surgical scissors and suspended on the chest wall by sutures for facilitating the surgical procedure. Two pacing leads were implanted onto the surface of the right ventricle and adjacent subcutaneous tissue beforehand, which was a vital safeguard procedure in case of emergency of malignant arrhythmia during operation. At last, SAN was destroyed by high-frequency electric knifes with energy of 60 J. The injection site was located at the right ventricle without fat covering. BMSCs-Tbx18 or BMSCs-GFP were implanted in the injection site (300 *μ*l per injection, 3 injections per pig, 4 × 10^6^ cells per injection).

### 2.9. Statistical Analysis

Data are expressed as the mean ± standard deviation, and statistical analysis was performed with GraphPad software. Normally distributed variables were analyzed using the Student *t*-test or paired *t*-test, and nonnormally distributed variables were analyzed using the Wilcoxon signed rank test. *P* value < 0.05 was considered statistically significant for all tests.

## 3. Results

### 3.1. Cell Characteristics of BMSCs and Demonstration of Gene Transfer

**Figure 1:**
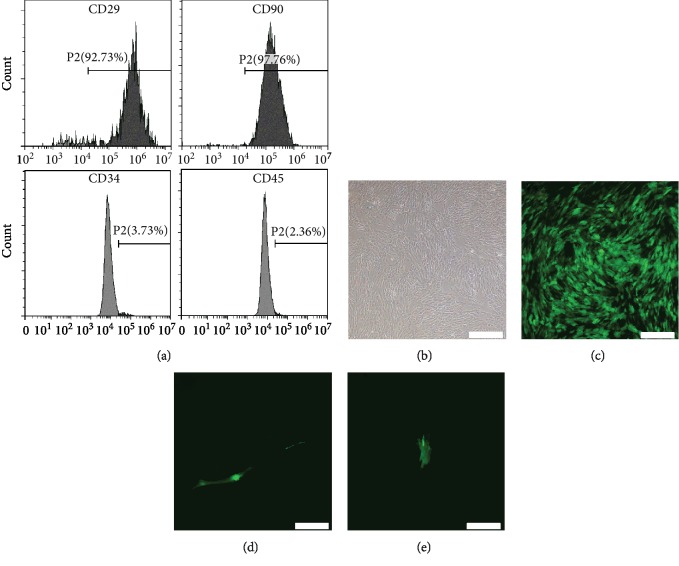
Cell characteristics of BMSCs and demonstration of gene transfer. (a) Surface marker of BMSCs under flow cytometry. BMSCs: bone mesenchymal stem cells; CD: cluster of differentiation. (b) Cellular morphological feature of BMSCs exhibiting a spindle shape. Scale bar = 400 *μ*m. (c) Green fluorescence of BMSCs after transfection with Lenti-Tbx18 for 48 h. Scale bar = 400 *μ*m. (d, e) Representative morphological characterizations of BMSCs with a strip shape and original spindle shape after transduction with Lenti-Tbx18 and Lenti-GFP for 7 days, respectively. Scale bar = 200 *μ*m.

Figure 1(a) shows the result of surface markers expressed in the isolated BMSCs, which are identified by flow cytometric analysis. The positive expression of stromal cell markers (CD29 and CD90) coupled with the absence of hematopoietic markers (CD45 and CD34) indicates that these cells were purified BMSCs. The result of negative control is shown in [Supplementary-material supplementary-material-1]. BMSCs exhibit spindle-like morphology after adherence as shown in Figure 1(b). When the cell confluence reached 80%, BMSCs were transfected with Lenti-Tbx18 and corresponding Lenti-GFP, respectively. Green fluorescence could be detected after transfection for 24 h (Figure 1(c)). In addition, optimal infection efficiency can be achieved when the multiple of infection (MOI) was set at 100. Figures 1(d) and 1(e) vividly show the morphological change of BMSCs after transfection for 7 days. BMSCs with an original spindle shape gradually transformed into a strip shape, which is a typical feature of SAN cells [22]. In addition, the beating was observed in approximately 7% BMSCs-Tbx18 at day 10 (approximately 90 bpm), and the beating could last for about 3 weeks *in vitro*.

### 3.2. Tbx18 Overexpression in BMSCs Promotes BMSCs Differentiation into Pacemaker-Like Cells

**Figure 2:**
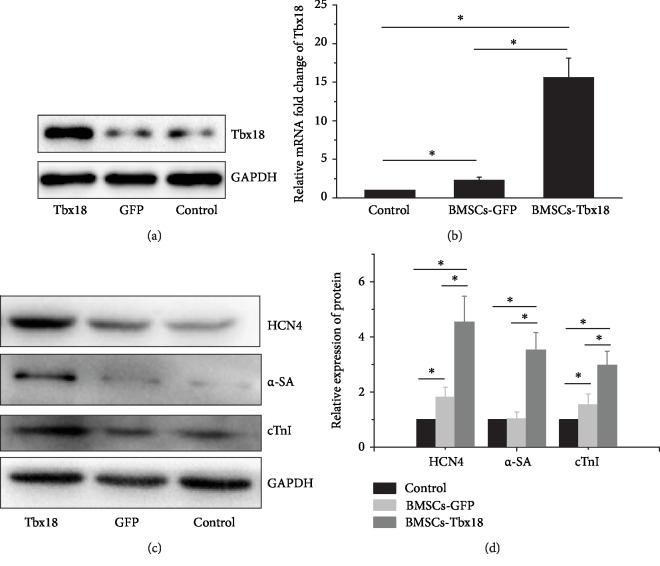
Western blotting and PCR analysis of target protein and mRNA expression level after transfection. (a) Western blotting of Tbx18 expression level after transfection for 72 h. BMSCs without transfection are set as the control. (b) PCR analysis of Tbx18 expression level after transfection for 48 h. ∗ represents *P* < 0.05. (c) Western blotting detects increased HCN4, *α*-SA, and cTnI protein expression. (d) Quantitative analysis of the protein expression levels of HCN4, *α*-SA, and cTnI. ∗ represents *P* < 0.05. cTnI: cardiac troponin I; GFP: green fluorescent protein; HCN4: hyperpolarization-activated cyclic nucleotide-gated channel 4; *α*-SA: *α*-striated actin.

The abovementioned morphological change suggests that BMSCs may differentiate into pacemaker-like cells after transduction by Lenti-Tbx18. While molecular evidence is also needed for further confirmation of such differentiation process. The expression level of Tbx18 is significantly upregulated, which is demonstrated by RT-qPCR and western blotting (Figures 2(a) and 2(b)). At the same time, the protein expression levels of *α*-SA, cTnI, and HCN4 were assessed by western blotting as shown in Figures 2(c) and 2(d). The results indicated that *α*-SA, cTnI, and HCN4 expression levels in the BMSC-Tbx18 group were significantly upregulated. Taking the morphological alteration and molecular change together, Tbx18 overexpression in BMSCs promotes BMSC differentiation into pacemaker-like cells.

### 3.3. Model Building of SAN Dysfunction in Pig

**Figure 3:**
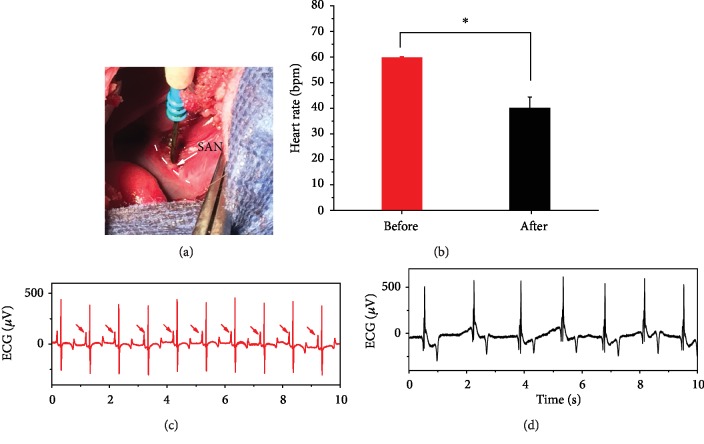
Model building of SAN dysfunction in pig. (a) Photograph of the construction procedure for SAN dysfunction by high-frequency electrotome. White arrow represents the location of SAN. (b) Mean heart rate of pigs before and after the model construction procedure. Data are means ± SEM. bpm: beats per minute. ∗ represents *P* < 0.05. (c) Representative ECG of sinus rhythm before the model construction procedure. Red arrow represents P wave. (d) Representative ECG of arrhythmia after the model construction procedure.

The SAN, which is located at the junction of the superior vena cava and the right atrium, sets the rhythm for the heart.  Figure 3(a) shows the photograph of the model building procedure for SAN dysfunction by a high-frequency electrotome. 9 pigs were used to construct SAN dysfunction model, 1 died of massive hemorrhage on account of superior vena cava rupture. Real-time ECG monitoring was necessary to assess the effect of model building. Normal ECG, including regular P waves, followed by QRS complex, and T waves, is shown in Figure 3(c). The mean heart rate (HR) is significantly reduced after the model building process (Figure 3(b)). The absence of P wave accompanied with arrhythmia (Figure 3(d)) verified that a SAN dysfunction model in large-scale animals can be successfully conducted by electrocoagulation using a surgical electrotome.

### 3.4. Tbx18 Biological Pacemaker Enhances Heart Rate with Favorable Autonomic Respondence

**Figure 4:**
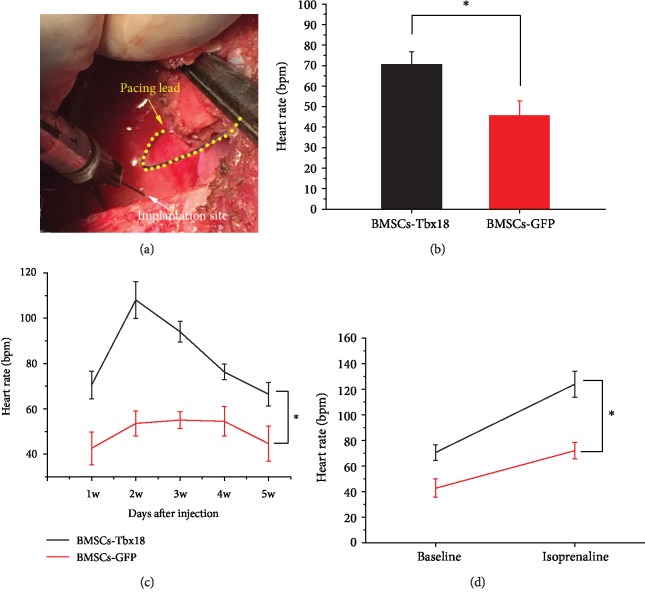
BMSCs-Tbx18 create a biological pacemaker. (a) Photograph of the implantation procedure for transduced cells. Yellow arrow represents the pacing lead of the temporary epicardial pacemaker, and white arrow represents the implantation site of the right ventricle. (b) Mean heart rate of the BMSC-Tbx18 group and BMSC-GFP group after injection for 2 days. Data are means ± SEM. ^∗^*P* < 0.05 compared to the BMSC-GFP group. (c) Mean heart rate of both groups after injection for continuous 5 weeks. Data are means ± SEM. ^∗^*P* < 0.05 compared to the BMSC-GFP group. (d) Increasement in heart rate after continuous isoproterenol infusion for 10 min between both two animal groups. ^∗^*P* < 0.05 compared to the BMSC-GFP group.

Figure 4(a) shows the implantation procedure of transduced BMSCs into the right ventricle. Meanwhile, a temporary pacemaker was employed to prevent the occurrence of malignant bradycardia arrhythmia and set as a positive control for cardiac pacing. The mean HR of the BMSC-Tbx18 group is significantly higher than that of the BMSC-GFP group in day 2 after injection and persisting for continuous 5 weeks as shown in Figures 4(b) and 4(c). In addition, the mean HR reaches its maximum value at the 2^nd^ week and then trends down. The HR of the BMSC-Tbx18 group maintains at a higher level than that of the BMSC-GFP group at all time points of our study. Since the poor engraftment and unwanted migration will inevitably occur during the implantation process, the immunofluorescence test was used to verify the existence of BMSCs-Tbx18 in the injection site at the end of the 5-week experiment. The positive expression of Tbx18 indicates that BMSCs-Tbx18 can be observed after 5 weeks as shown in [Supplementary-material supplementary-material-1].  Figure 4(d) shows that isoproterenol infusion (3 *μ*g/kg/h) for 10 min increased HR by 72% and 65% in the BMSC-Tbx18 group and BMSC-GFP group, respectively. The active reaction to the *β*-adrenergic agonist indicates that the Tbx18 biological pacemaker may provide an avenue to address the shortcoming of insufficient response to neurotransmitter signals for the electronic pacemaker.

### 3.5. BMSC-Tbx18 Biological Pacemaker Activity Originates from the Right Ventricle

**Figure 5:**
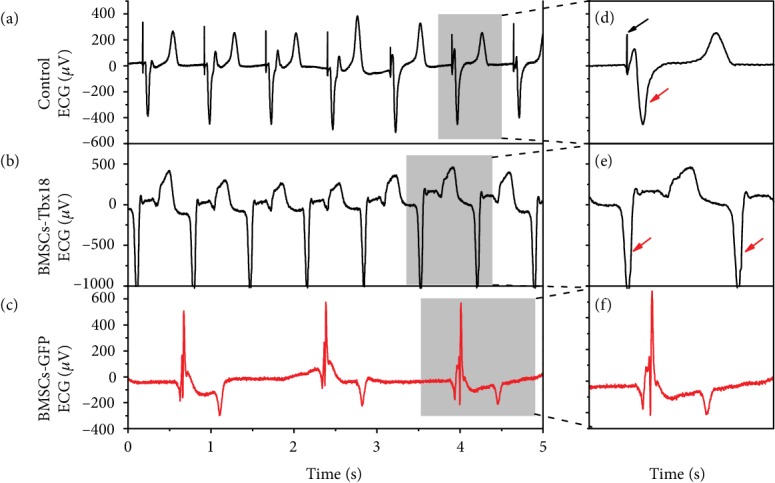
BMSC-Tbx18 biological pacemaker activity originates from the right ventricle. (a–c) Representative ECG of the temporary epicardial pacemaker, BMSC-Tbx18 group, and BMSC-GFP group; the pacing rate of the epicardial pacemaker was preset at 80 bpm as a control. (d–f) Enlarged figure of corresponding ECG for the temporary epicardial pacemaker, BMSC-Tbx18 group, and BMSC-GFP group; red arrows represent the prolonged QRS complex (duration > 0.12 s), and black arrow represents the ventricular pacer spike.

To investigate the origin site of cardiac electrical activity, we use the DAQ system to capture and analyze ECG instantaneously. As we know, the electronic pacemaker is fabricated to stimulate the contraction of ventricles by simulating the function of SAN through releasing electrical stimuli periodically. Cardiac pacing can be achieved by inserting a pacing lead into the right ventricular endocardium intravenously for permanent application or suturing a pacing lead on the surface of the right ventricle for temporary use. Figures 5(a) and 5(d) show typical ECG of electronic pacing, in which a pacer spike followed by a prolonged QRS complex can be observed. Regular prolonged QRS complexes without corresponding pacer spike in the BMSC-Tbx18 group indicated that pacing signals were derived from the right ventricle (Figures 5(b) and 5(e)). In contrast, no ectopic electrical signal was observed in the BMSC-GFP group (Figures 5(c) and 5(f)). Together, these results support that Tbx18-induced pacemaker activity was released from the right ventricle.

## 4. Discussion

Previous studies have proposed the notion that Tbx18 could significantly enhance the converting efficiency of mature cardiac myocytes into pacemaker-like cells. Notably, the porcine cardiomyocytes were demonstrated to be transformed into pacemaker-like cells morphologically and functionally, in which the ectopic pacing site can maintain their function for nearly 2 weeks [14]. However, this issue remains controversial since no aberrant expression of SAN-specific genes could be observed in the chamber myocardium of fetal mice after Tbx18 reexpression [23]. In addition, pathological structural abnormalities, including right ventricular hypoplasia, atrial dilatation, and ventricular septal defects, were observed in this procedure. The induced pacemaker-like cells have been reported to be with high cAMP level, which is related to their function while they may affect numerous genes regulated by cAMP-dependent phosphorylation, leading to undesirable effects like neoplasm [24, 25].

In this article, we successfully create a biological pacemaker with longer operating time by modifying porcine BMSCs with Lenti-Tbx18 and verify its potential in the porcine SAN dysfunction model, which is consistent with the previous reports that Tbx18 can be used as a candidate for biological pacing.

The expression level of HCN is significantly upregulated after transduction of Tbx18, which is identical to previous results and may be related to aberrant pacemaker-like cell formation [15, 16, 26, 27]. It is reported that autodepolarization of SAN cells at hyperpolarization is due to the funny current (*I*_f_), which is driven by HCN protein [7, 28, 29]. HCN mutations may be associated with multiple families of bradycardia and SAN dysfunction, which indicates that HCN may play a vital role in maintaining stable function of SAN [30, 31]. Previous animal experiments have demonstrated that overexpression of HCN4 in the porcine heart can significantly reduce the dependence on pacemakers; furthermore, an ectopic pacing region can be induced by implanting BMSCs loaded with HCN4 [9, 32–34].

In addition, several procedures to construct a SSS animal model have been introduced in previous research, primarily involving physical impairment of the SAN, including condensation, radiofrequency ablation, and ligation of the right coronary artery. While in this paper, we successfully construct a model of SAN dysfunction by surgical technique under direct vision with a higher achievement ratio. However, a pig died of massive hemorrhage since the superior vena cava was ruptured by electrocoagulation, which suggests that a safer model building method should be further explored. Sodium hydroxide or formaldehyde wet compression has been successfully applied in small animal models with lower death rate, which may provide some hints for model building in large-scale animals [35–37]. A combination of physical and chemical methods may provide a new model building way with a higher achievement ratio and lower rate of complications.

In conclusion, we successfully create a biological pacemaker with longer operating time by modifying porcine BMSCs with Lenti-Tbx18 and verify its potential in the porcine SAN dysfunction model. This study may provide a novel alternative treatment for bradyarrhythmia. However, Tbx18 overexpression may lead to some unexpected complications, which requires further research in large-scale animals prior to application in clinical practice.

## Figures and Tables

**Table 1:** Primer sequences used in the study.

Gene	Primer sequences
Tbx18	Forward: ACCCTCAACCGATACAGCAC
	Reverse: GACATTCCCGAAATCTGCAT
GAPDH	Forward: GAGTCAACGGATTTGGTCGT
	Reverse: TTGATTTTGGAGGGATCTCG

## Data Availability

The original data used to support the findings of this study are available from the corresponding author upon request.

## Supplementary Materials

Supplementary Materials Figure S1: the flow cytometry histograms of the negative control group. Figure S2: laser confocal microscopy of immunofluorescence staining for Tbx18 in the rejection site. (A) Confocal microscopy image showing positive expression of TBX18 (green fluorescence). 4′,6-diamidino-2-phenylindole (DAPI) indicates nuclei. Scale bar, 50 *μ*m.
