# Supplementary material for: Genetically Modified Porcine Mesenchymal Stem Cells by Lentiviral Tbx18 Create a Biological Pacemaker
Source: Stem Cells Int. 2019 Nov 7;2019:3621314. doi: 10.1155/2019/3621314 (PMC6877911; doi:10.1155/2019/3621314)
Supplement: Supplementary Materials — Figure S1: the flow cytometry histograms of the negative control group. Figure S2: laser confocal microscopy of immunofluorescence staining for Tbx18 in the rejection site. (A) Confocal microscopy image showing positive expression of TBX18 (green fluorescence). 4′,6-diamidino-2-phenylindole (DAPI) indicates nuclei. Scale bar, 50 μm. [file 3621314.f1.docx]

**Supplementary materials**

Genetically modified porcine mesenchymal stem cells by lentiviral

Tbx18 create biological pacemaker

Yannan Hu^1#^, Ning Li ^1#^, Liang Liu^1,2#^, Hao Zhang^1^, Xiang Xue^3^, Xin Shao^1^, Yu Zhang^1^, Xilong Lang^1＊^

**Author affiliations**

^1^ Department of Cardiothoracic Surgery, Changhai Hospital, Second Military Medical University, Shanghai, China

^2^ Department of Thoracic Surgery, Changzhou TCM Hospital, Changzhou, China

^3^ Department of Cardiothoracic Surgery, The Affiliated Second Hospital, Suzhou University, Suzhou, China

# Yannan Hu, Ning Li, and Liang Liu contributed equally to this work.

**Correspondence**

* Xilong Lang, Department of Cardiothoracic Surgery, Changhai Hospital, Second Military Medical University, Shanghai, China.

Email: [langxl2017@163.com](mailto:langxl2017@163.com)





**Figure S1** the result of flow cytometry histograms in negative control group.





**Figure S2** (A) Confocal microscopy image showing positive expression of TBX18 (green fluorescence). 4′,6-diamidino-2-phenylindole (DAPI) indicates nuclei. Scale bar, 50 μm.
